# Prevalence and Features of Thyroglossal Duct Cyst on Ultrasonography, According to Radioactive Iodine Therapy: A Single-Center Study

**DOI:** 10.3389/fendo.2020.00188

**Published:** 2020-04-03

**Authors:** Ji Sun Park, Dong Wook Kim, Gi Won Shin, Jin Young Park, Yoo Jin Lee, Hye Jung Choo, Ha Kyoung Park, Tae Kwun Ha, Do Hun Kim, Soo Jin Jung, Sung Ho Moon, Ki Jung Ahn, Hye Jin Baek

**Affiliations:** ^1^Department of Nuclear Medicine, Busan Paik Hospital, Inje University College of Medicine, Busan, South Korea; ^2^Department of Radiology, Busan Paik Hospital, Inje University College of Medicine, Busan, South Korea; ^3^Department of General Surgery, Busan Paik Hospital, Inje University College of Medicine, Busan, South Korea; ^4^Department of Otorhinolaryngology-Head and Neck Surgery, Busan Paik Hospital, Inje University College of Medicine, Busan, South Korea; ^5^Department of Pathology, Busan Paik Hospital, Inje University College of Medicine, Busan, South Korea; ^6^Department of Anesthesiology and Pain Medicine, Busan Paik Hospital, Inje University College of Medicine, Busan, South Korea; ^7^Department of Radiation Oncology, Busan Paik Hospital, Inje University College of Medicine, Busan, South Korea; ^8^Department of Radiology, Gyeongsang National University Changwon Hospital, Gyeongsang National University School of Medicine, Changwon, South Korea

**Keywords:** thyroglossal duct cyst, thyroidectomy, radioactive iodine ablation, ultrasonography, prevalence

## Abstract

**Objective:** The relationship between radioactive iodine therapy (RIT) and prevalence of thyroglossal duct cysts (TGDC) on ultrasonography (US) has not been reported. We assessed the prevalence and US features of TGDC according to RIT.

**Methods:** From July 2017 to June 2018, 3,146 subjects underwent thyroid or neck US at our center. The presence or absence of TGDCs was prospectively investigated based on real-time US examination. Among the 3,146 subjects, 261 subjects were excluded because of <18 years of age, unclear information of RIT, or the presence of a radiation therapy history to the neck. Eventually, 2,885 subjects were included in this study.

**Results:** Of the 2,885 subjects finally included, 126 (4.4%) showed a TGDC on US. Those with RIT history showed a higher prevalence of TGDCs than those without (no statistical difference, *p* = 0.062). In 697 male subjects, there were statistical differences in type of surgery, RIT history, and session number of RIT between those with or without TGDCs (*p* < 0.0001). In 126 subjects with TGDCs, only sex showed a significant difference between those with or without RIT history (*p* = 0.015). However, there were no significant differences in the location, size, and shape of TGDCs (*p* > 0.05). The common US features of TGDC were suprahyoid location, ~1 centimeter, and flat-to-ovoid or round shape.

**Conclusions:** RIT may increase the prevalence of TGDCs, particularly in men.

## Introduction

Thyroglossal duct cysts (TGDCs) are the most common congenital neck mass, usually found centrally, and account for 70% of congenital neck anomalies (1). The thyroglossal duct originates during embryonic development of the thyroid gland, and typically involutes and atrophies after migration of the primitive thyroid to its final position in the inferior neck (1). Failure of involution and atrophy results in persistence of a thyroglossal duct remnant (1). Approximately 7% of the general population has a thyroglossal duct remnant (2, 3). If any portion of the thyroglossal duct persists, secretions from the epithelial lining may give rise to cystic lesions—TGDCs—sized from 0.5 cm to 6 cm in diameter, with most being between 1.5 cm and 3 cm (1). One study reported that asymptomatic enlargement of pre-existing TGDCs can occur after radiation therapy to the neck, in head and neck cancer patients (4). These authors proposed that inflammation associated with radiation therapy may result in secretory stimulation and/or obstruction of the thyroglossal duct remnant.

Radioactive iodine therapy (RIT) using iodine-131 (I-131) has been a commonly accepted procedure for ablation of remnant thyroid tissue after thyroidectomy in patients with differentiated thyroid cancer. Radioactive iodine localizes in the remnant thyroid tissue through the sodium-iodide symporter in thyroid follicular cells (5, 6). Several previous studies reported visualization of the thyroglossal duct remnant on radioiodine whole-body scan in patients with differentiated thyroid cancer after thyroidectomy (7, 8). To our knowledge, no previous studies have investigated the relationship between RIT and prevalence of TGDC on US. We therefore aimed to assess the prevalence and US features of TGDC according to RIT using a retrospective cohort study design.

## Methods

### Study Population

From July 2017 to June 2018, 3,146 subjects underwent thyroid or neck US examination at the Thyroid and Head & Neck Cancer Center of our hospital. Among them, 261 subjects were excluded owing to their youth (<18 years; *n* = 238), unclear information on RIT (*n* = 20), and a history of radiation therapy to the neck (*n* = 3). Ultimately, 2,885 subjects (2,188 female and 697 male; mean age ± standard deviation (SD): 54.1 ± 11.0 years; age range: 18–86 years) were included in this study. The study was approved by the Busan Paik Hospital Institutional Review Board (IRB 19-0152), and the need for written informed consent was waived owing to the retrospective analysis.

### Ultrasonographic Examination and Image Analysis

Thyroid or neck ultrasonography (US) was performed by one experienced radiologist (with 17 years of experience in performing thyroid or neck US) using a high-resolution ultrasound scanner (HDI 5000 or iU 22, Philips Medical Systems, Bothell, WA, USA; and Aplio 400, Toshiba Medical Systems, Tokyo, Japan) with a 5–12-MHz linear probe. One of these two US instruments was randomly selected for each subject. To evaluate a subject for a TGDC, the radiologist investigated the midline neck from tongue base to thyroid isthmus, using real-time thyroid or neck US. This was a routine part of thyroid or neck US, and did not impose any time constraints or economic burden on the subjects.

The radiologist prospectively determined the presence or absence of a TGDC using real-time US, with TGDC defined as a thin-walled, anechoic cyst, typically located under the strap muscle and with no vascularity in the anterior upper midline neck (9). TGDCs of <3 mm at greatest diameter were excluded from this study. The same radiologist retrospectively investigated US features of TGDC using a picture archiving communication system; such features included the location (suprahyoid and infrahyoid), largest diameter, and shape (flat to ovoid, round, tubular, or amorphous).

### Radioactive Iodine Therapy

After thyroidectomy, RIT was performed using either traditional thyroid hormone withdrawal or recombinant human thyrotropin-stimulated preparation methods to ensure adequate stimulation of thyroid-stimulating hormone. Every patient had previously been treated with thyroidectomy for differentiated thyroid cancer. In the group that underwent traditional thyroid hormone withdrawal, levothyroxine was withdrawn for at least 3 weeks before RIT. The recombinant human thyrotropin-stimulated group received 0.9 mg of recombinant human thyrotropin (Thyrogen; Genzyme Corporation, Cambridge, MA, USA) intramuscularly on days 1 and 2, with RIT administered on day 3. The patients were advised to maintain a low-iodine diet for at least 1 week prior to administration of radioactive iodine. The therapeutic dose of I-131 for each treatment ranged from 1.1 GBq (30 mCi) to 7.4 GBq (200 mCi).

### Statistical Analysis

The data were tested for normal distribution and normal distribution of continuous data with the Kolmogorov-Smirnov test. Normally distributed variables were compared using the independent *t*-test, and expressed as mean ± SD. Comparisons of categorical variables in each group were performed using the χ^2^ test or, for small cell values, Fisher's exact test. All statistical analyses were conducted using SPSS, Version 24.0 (IBM, Armonk, New York, USA). A *P* value < 0.05 was regarded as statistically significant.

## Results

During the study period, 2,885 subjects underwent thyroid or neck US examination for various clinical reasons: postoperative follow-up (*n* = 1595), preoperative staging (*n* = 52), health screening (*n* = 790), anterior neck discomfort (*n* = 10), abnormal thyroid or parathyroid serology (*n* = 80), patient request (*n* = 9), US follow-up of known thyroid nodules (*n* = 324), further evaluation of known thyroid nodules or lymph nodes seen on computed tomography or magnetic resonance imaging (*n* = 328), and palpable neck masses (*n* = 13). No subject underwent two or more sessions of thyroid or neck US. Of the 2,885 subjects, 1,593 (55.2%) had a history of previous thyroid surgery; total thyroidectomy was performed in 981 patients (61.6%), hemithyroidectomy in 606 (38.0%), and isthmusectomy in 6 (0.4%). Among the patients who underwent total thyroidectomy, 545 (55.6%) had one or more sessions of RIT (mean 1.1 ± 0.3, range: 1–3). The highest dose of RIT ranged from 30 to 200 mCi (mean ± SD: 128.7 ± 34.0 mCi); the highest dose was 30 mCi in 21 patients, 60 mCi in 2, 80 mCi in 7, 100 mCi in 185, 130 mCi in 42, 150 mCi in 209, 160 mCi in 37, 180 mCi in 40, and 200 mCi in 2 patients.

**Figure 1:**
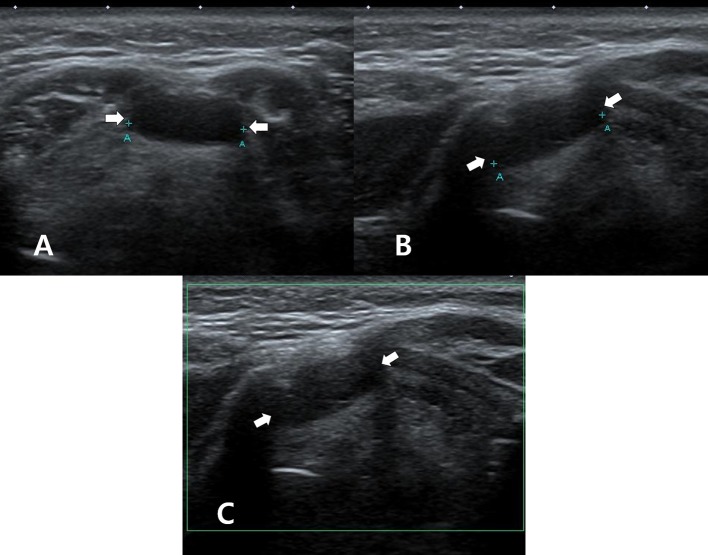
A 50~55-year-old woman who underwent thyroid ultrasonography (US) owing to health screening but had no history of thyroid surgery or radioactive iodine ablation. On thyroid US, transverse gray-scale **(A)**, longitudinal gray-scale **(B)**, and longitudinal color Doppler **(C)** US images show a well-defined cystic lesion with a flat-to-ovoid shape in the suprahyoid, anterior upper midline neck (arrows, 1.3 cm in length).

**Figure 2:**
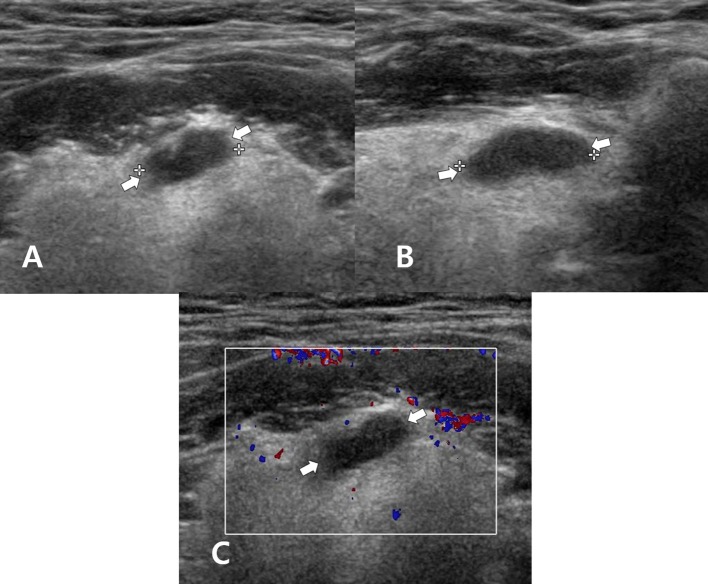
A 60~65-year-old man who underwent total thyroidectomy and one session of radioactive iodine ablation (160 mCi) for papillary thyroid carcinoma. On the follow-up neck ultrasonography (US), transverse gray-scale **(A)**, longitudinal gray-scale **(B)**, and transverse color Doppler **(C)** US images show a well-defined cystic lesion with a flat-to-ovoid shape in the suprahyoid, anterior upper midline neck (arrows, 1.3 cm in length).

Of the 2885 subjects, 126 (4.4%) exhibited a TGDC on US (mean size: 9.0 ± 4.0 mm, range: 3–33.0 mm) (Figures 1, 2). Clinical and US findings in the 2,885 subjects according to presence or absence of TGDCs are compared in Table 1. The group with a history of RIT showed a higher prevalence of TGDCs than those without, but no statistical difference was found (*p* = 0.062). There were no significant differences in age, gender, reason for thyroid/neck US, type of thyroid surgery, and session number of RIT, between those with TGDC and those without (p > 0.05). In the 697 male subjects, the clinical and US findings were compared according to the presence or absence of TGDCs (Table 2). Statistical differences between the groups with or without TGDCs were seen for type of surgery, RIT history, and session number of RIT (*p* < 0.0001).

**Table 1:** Comparison of clinical and ultrasonographic findings in all 2,885 subjects, according to presence or absence of thyroglossal duct cysts on ultrasonography.

**Items**	**TGDC (+)** **(*n* = 126)**	**TGDC (–)** **(*n* = 2759)**	***p* value**
Age (mean ± SD, yr)	52.3 ± 10.8	54.1 ± 11.0	0.054
Gender			0.925
Female	96 (76.2)	2092 (75.8)	
Male	30 (23.8)	667 (24.2)	
Reason for thyroid/neck US			0.261
Postoperative follow-up	76 (60.3)	1519 (95.1)	
Preoperative staging	4 (3.2)	48 (1.7)	
Health screening	34 (27)	756 (27.4)	
Anterior neck discomfort	0	10 (0.4)	
Abnormal thyroid/parathyroid serology	6 (4.8)	74 (2.7)	
Patient request	0	9 (0.3)	
US follow-up of known thyroid nodule	6 (4.8)	318 (11.5)	
Known thyroid nodule or lymph node in CT or MRI	0	12 (0.4)	
Palpable neck mass	0	13 (0.5)	
Type of thyroid surgery			0.111
No thyroid surgery	50 (39.7)	1242 (45)	
Total thyroidectomy	52 (41.3)	929 (33.7)	
Hemithyroidectomy	23 (18.3)	583 (21.1)	
Isthmusectomy	2 (0.8)	5 (0.2)	
Nodulectomy	0	0	
Application of RIT			0.062
No	94 (74.6)	2246 (81.4)	
Yes	32 (25.4)	513 (18.6)	
Session number of RIT			0.104
0	94 (74.6)	2246 (81.4)	
1	28 (22.2)	472 (17)	
2	4 (3.2)	40 (1.4)	
3	0	1 (0.1)	

*Data are number of items, with percentage in parentheses. TGDC, thyroglossal duct cyst; SD, standard deviation; US, ultrasonography; CT, computed tomography; MRI, magnetic resonance imaging; RIT, radioactive iodine therapy; NA, not applicable*.

**Table 2:** Comparison of clinical and ultrasonographic findings in the 697 male subjects, according to presence or absence of thyroglossal duct cysts on ultrasonography.

**Items**	**TGDC (+)** **(*n* = 30)**	**TGDC (–)** **(*n* = 667)**	***p* value**
Age (mean ± SD, yr)	53.8 ± 11.1	53.6 ± 11.2	0.916
Reason for thyroid/neck US			0.549
Postoperative follow-up	16 (53.3)	223 (33.4)	
Preoperative staging	0	10 (1.5)	
Health screening	12 (40)	338 (50.7)	
Anterior neck discomfort	0	2 (0.3)	
Abnormal thyroid/parathyroid serology	1 (3.3)	19 (2.8)	
Patient request	0	1 (0.1)	
US follow-up of known thyroid nodule	1 (3.3)	62 (9.3)	
Known thyroid nodule in CT or MRI	0	3 (0.4)	
Palpable neck mass	0	7 (1)	
PET (+) L/N	0	2 (0.3)	
Type of thyroid surgery			<0.0001
No thyroid surgery	14 (46.7)	444 (66.6)	
Total thyroidectomy	16 (53.3)	141 (21.1)	
Hemithyroidectomy	0	81 (12.1)	
Isthmusectomy	0	1 (0.1)	
Nodulectomy	0	0	
Application of RIT			<0.0001
No	17 (56.7)	565 (84.7)	
Yes	13 (43.3)	102 (15.3)	
Session number of RIT			<0.0001
0	17 (56.7)	565 (84.7)	
1	10 (33.3)	95 (14.2)	
2	3 (10)	7 (1)	
3	0	0	

*Data are number of items, with percentage in parentheses. TGDC, thyroglossal duct cyst; SD, standard deviation; US, ultrasonography; CT, computed tomography; MRI, magnetic resonance imaging; RIT, radioactive iodine therapy; NA, not applicable*.

Comparison of clinical and US findings in the 126 subjects exhibiting TGDCs, according to the presence or absence of radioactive iodine ablation therapy, are shown in Table 3. Among these 126 subjects, 32 underwent one or more sessions of RIT after total thyroidectomy, whereas 94 did not undergo RIT. The mean size of TGDCs in those with or without a history of RIT was 9.5 ± 4.5 mm and 8.5 ± 4.4 mm, respectively. There was a significant difference in gender (*p* = 0.015) between the group with a history of RIT and those without. However, there were no significant differences in age, reason for thyroid/neck US, type of thyroid surgery, and location, size, or shape of TGDCs (*p* > 0.05). The common US features of TGDC were suprahyoid location, diameter of ~1 cm, and flat-to-ovoid or round shape.

**Table 3:** Comparison of clinical and ultrasonographic findings in the 126 subjects with thyroglossal duct cysts on ultrasonography, according to presence or absence of radioactive iodine ablation therapy history.

**Items**	**RIT (+)** **(*n* = 32)**	**RIT (–)** **(*n* = 94)**	***p* value**
Age (mean ± SD, yr)	52.5 ± 10.6	52.2 ± 10.9	0.882
Gender			0.015
Female	19 (59.4)	77 (81.9)	
Male	13 (40.6)	17 (18.1)	
Reason for thyroid/neck US			<0.0001
Postoperative follow-up	32 (100)	44 (46.8)	
Preoperative staging	0	4 (4.3)	
Health screening	0	34 (36.2)	
Anterior neck discomfort	0	0	
Abnormal thyroid/parathyroid serology	0	6 (6.4)	
Patient request	0	0	
US follow-up of known thyroid nodule	0	6 (6.4)	
Known thyroid nodule or lymph node in CT or MRI	0	0	
Palpable neck mass	0	0	
Type of thyroid surgery			<0.0001
No thyroid surgery	0	50 (53.2)	
Total thyroidectomy	32 (100)	20 (21.3)	
Hemithyroidectomy	0	23 (24.5)	
Isthmusectomy	0	1 (1.1)	
Nodulectomy	0	0	
Location of TGDC on US			1.000
Suprahyoid	28 (87.5)	82 (87.2)	
Infrahyoid	4 912.5)	12 (12.8)	
Size of TGDC (mean ± SD, mm)	9.5 ± 4.5	8.5 ± 4.4	0.277
Shape of TGDC			0.703
Round	11 (34.4)	35 (37.2)	
Tubular	2 (6.3)	3 (3.2)	
Amorphous	2 (6.3)	11 (11.7)	
Flat-to-ovoid	17 (53.1)	45 (47.9)	

*Data are number of items, with percentage in parentheses. TGDC, thyroglossal duct cyst; SD, standard deviation; US, ultrasonography; CT, computed tomography; MRI, magnetic resonance imaging; RIT, radioactive iodine therapy; NA, not applicable*.

Comparison of clinical and US findings in the 545 subjects who underwent RIT, according to the presence or absence of TGDC, is shown in Table 4. There was a significant difference in gender between the group with TGDC and that without (*p* = 0.005). However, there were no significant differences in age, maximum dose of RIT, and interval between RIT and US (*p* > 0.05).

**Table 4:** Comparison of clinical and ultrasonographic findings in the 545 subjects who underwent radioactive iodine therapy, according to presence or absence of the thyroglossal duct cyst.

**Items**	**TGDC (+)** **(*n* = 32)**	**TGDC (–)** **(*n* = 513)**	***p* value**
Age (mean ± SD, yr)	52.5 ± 10.6	54.2 ± 11.4	0.416
Gender			0.005
Female	19 (59.4)	411 (80.1)	
Male	13 (40.6)	102 (19.9)	
Maximum dose of radioactive iodine (mean ± SD, mCi)	118.1 ± 34.3	129.4 ± 33.9	0.07
Interval between RIT and US (mean ± SD, month)	63.4 ± 32.6	70.3 ± 38.0	0.313

*Data are number of items, with percentage in parentheses. TGDC, thyroglossal duct cyst; SD, standard deviation; US, ultrasonography; RIT, radioactive iodine therapy; NA, not applicable*.

## Discussion

Our study findings revealed that RIT may increase the prevalence of TGDCs, particularly in male subjects, but may not influence the location, size, and shape of TGDCs. Although TGDC is typically recognized in pediatric patients, it has also been known to present in adults, with varying frequency (10–12). The prevalence of TGDC varies, based on factors such as study population and detection methods. In a previous microscopic examination of 200 adult larynges, TGDCs were observed in 7% of specimens (3). In a cadaver study, 15% of 80 adult cadavers had a TGDC (13). However, in one previous study using US, 513 of 54,369 asymptomatic adults (0.9%) showed TGDC ≥3 mm at the greatest diameter, and most TGDCs (77.3%) showed no interval change in size (12). In our study, 126 (4.4%) subjects showed a TGDC on US, a higher rate than that in the previous study, despite use of the same criterion for size (≥ 3 mm at largest diameter). The reason for the high prevalence of TGDCs on US in our study is unclear; however, the close relationship between RIT and TGDCs should be considered. In particular, male subjects showed a significantly higher prevalence of TGDCs in the group with a history of RIT. Therefore, we believe that RIT increases the prevalence of TGDCs, particularly in male subjects.

Several previous case reports showed newly developed or enlarged TGDCs after radiation therapy in head and neck cancer patients. The authors suggested that radiation-induced local inflammation may contribute to cyst formation in the thyroglossal duct remnant (4, 14). It has been recognized that TGDC is lined by squamous or respiratory epithelium, and secretions from the epithelial lining may give rise to cystic lesions (1, 3, 10). To our knowledge, however, no previous studies have investigated the relationship between RIT and size of TGDCs. Our study found no significant association between RIT and size of TGDCs. The reason for this difference is unclear. However, the action mechanism of radiation therapy and RIT may be different depending on the size of TGDCs. To clarify this issue, further studies are required.

In the literature, TGDCs present a purely anechoic mass with posterior enhancement and without any internal septa or solid components on US, and are typically embedded in strap muscle (9, 12). The same definition of TGDCs was adopted in our study. In the present study, the common US features of TGDC were suprahyoid location, diameter of ~1 cm, and flat-to-ovoid or round shape. These findings are similar to those of previous studies (12).

Several limitations to this study should be considered. Firstly, pathological confirmation of TGDCs was not performed. Furthermore, TGDCs of <3 mm at their largest diameter were not included. Secondly, young subjects (<18 years) were not included, and neither were 20 subjects with unclear information on RIT. Thus, selection bias should be considered. Thirdly, the interval between RIT and US examination varied. Finally, US features of TGDCs were investigated retrospectively using limited US images.

In conclusion, RIT may increase the prevalence of TGDCs, particularly in male subjects. However, RIT may not influence the location, size, and shape of TGDCs.

## Data Availability Statement

The raw data supporting the conclusions of this article will be made available by the authors, without undue reservation, to any qualified researcher.

## Ethics Statement

The studies involving human participants were reviewed and approved by Busan Paik Hospital (IRB-19-0152). Written informed consent for participation was not required for this study in accordance with the national legislation and the institutional requirements.

## Author Contributions

DWK: concept and design and review of final manuscript. JSP and DWK: acquisition of data and manuscript writing. HB and DWK: analysis and interpretation of data. All authors: literature review and refinement of manuscript.

### Conflict of Interest

The authors declare that the research was conducted in the absence of any commercial or financial relationships that could be construed as a potential conflict of interest.

## References

[1] Koeller KK Alamo L Adair CF Smirniotopoulos JG. Congenital cystic masses of the neck: radiologic-pathologic correlation. Radiographics. (1999) 19:152–3. 10.1148/radiographics.19.1.g99ja06121 9925396

[2] Carter Y Yeutter N Mazeh H. Thyroglossal duct remnant carcinoma: beyond the Sistrunk procedure. Surg Oncol. (2014) 23:161–6. 10.1016/j.suronc.2014.07.002 25056924 PMC4149934

[3] Thompson LD Herrera HB Lau SK. A clinicopathologic series of 685 thyroglossal duct remnant cysts. Head Neck Pathol. (2016) 10:465–74. 10.1007/s12105-016-0724-7 27161104 PMC5082048

[4] Singh S Rosenthal DI Ginsberg LE. Enlargement and transformation of thyroglossal duct cysts in response to radiotherapy: imaging findings. AJNR Am J Neuroradiol. (2009) 30:800–2. 10.3174/ajnr.A1448 19131415 PMC7051746

[5] Chudgar AV Shah JC. Pictorial review of false-positive results on radioiodine scintigrams of patients with differentiated thyroid cancer. Radiographics. (2017) 37:298–315. 10.1148/rg.2017160074 28076008

[6] Silberstein EB Alavi A Balon HR Clarke SE Divgi C Gelfand MJ. The SNMMI practice guideline for therapy of thyroid disease with 131I 3.0. J Nucl Med. (2012) 53:1633–51. 10.2967/jnumed.112.105148 22787108

[7] Lee SW Lee J Lee HJ Seo JH Kang SM Bae JH. Enhanced scintigraphic visualization of thyroglossal duct remnant during hypothyroidism after total thyroidectomy: prevalence and clinical implication in patients with differentiated thyroid cancer. Thyroid. (2007) 17:341–6. 10.1089/thy.2006.0274 17465864

[8] Mohamed Sayed MH Saleh Farghaly HR Fadl FA. Rate of thyroglossal duct remnant visualization after total thyroidectomy for differentiated thyroid carcinoma and its impact on clinical outcome of radioactive iodine (I-131) ablation. Indian J Nucl Med. (2015) 30:116–21. 10.4103/0972-3919.152970 25829728 PMC4379669

[9] Ahuja AT King AD King W Metreweli C Thyroglossal duct cysts: sonographic appearance in adults. AJNR Am J Neuroradiol. (1999) 20:579–82. 10319964 PMC7056022

[10] Ewing CA Kornblut A Greeley C Manz H. Presentations of thyroglossal duct cysts in adults. Eur Arch Otorhinolaryngol. (1999) 256:136–8. 10.1007/s004050050126 10234482

[11] Yim MT Tran HD Chandy BM. Incidental radiographicfindings of thyroglossal duct cysts: Prevalence and management. Int J Pediatr Otorhinolaryngol. (2016) 89:13–6. 10.1016/j.ijporl.2016.07.024 27619021

[12] Kim SC Sun HY Kim HS Ryoo I. Long-term ultrasound follow-up of incidentally detected thyroglossal duct cysts in adults. AJNR Am J Neuroradiol. (2018) 39:2356–2359. 10.3174/ajnr.A5882 30467213 PMC7655380

[13] Kurt A Ortug C Aydar Y Ortug G. An incidence study on thyroglossal duct cysts in adults. Saudi Med J. (2007) 28:593–7. 17457484

[14] Srinivasan A Hayes M Chepeha D Mukherji SK. Rare presentation of thyroglossal duct cyst after radiation therapy to the neck. Australas Radiol. (2007) 51:B180–2. 10.1111/j.1440-1673.2007.01842.x 17991058

